# The COVID-19 pandemic and temporal change in metabolic risk factors for cardiovascular disease: A natural experiment within the HELIUS study

**DOI:** 10.1016/j.ssmph.2023.101432

**Published:** 2023-05-19

**Authors:** Bryn Hummel, Mara A. Yerkes, Ralf E. Harskamp, Henrike Galenkamp, Anton E. Kunst, Anja Lok, Irene G.M. van Valkengoed

**Affiliations:** a Department of Public and Occupational Health, Amsterdam Public Health Research Institute, Amsterdam UMC, University of Amsterdam, Meibergdreef 9, 1105, AZ, Amsterdam, the Netherlands; b Department of Interdisciplinary Social Sciences, Utrecht University, Heidelberglaan 8, 3584, CS, Utrecht, the Netherlands; c Department of General Practice, Amsterdam UMC, University of Amsterdam, Meibergdreef 9, 1105, AZ, Amsterdam, the Netherlands; d Department of Psychiatry, Amsterdam University Medical Centre, Meibergdreef 9, 1105, AZ, Amsterdam, the Netherlands

**Keywords:** Cardiovascular disease, Cardiometabolic health, COVID-19, Ethnic differences, Sex differences, The HELIUS study

## Abstract

The coronavirus disease 2019 (COVID-19) pandemic, including the restrictive measures taken to reduce the spread of the virus, negatively affected people's health behavior. We explored whether the pandemic also had an effect on metabolic risk factors for cardiovascular disease (CVD) in women and men. We conducted a natural experiment, using data from 6962 participants without CVD at baseline (2011–2015) of six ethnic groups of the HELIUS study in Amsterdam, the Netherlands. We studied whether participants whose follow-up measurements were taken within the 11 months before the pandemic (control group) differed from those whose measurements were taken taken within 6 months after the first lockdown (exposed group). Using sex-stratified linear regressions with inverse probability weighting, we compared changes in baseline- and follow-up data between the control and exposed group in six metabolic risk factors: systolic and diastolic blood pressure (SBP, DBP), total cholesterol (TC), fasting plasma glucose (FPG), hemoglobin A1c (HbA1c), and estimated glomerular filtration rate (eGFR). Next, we explored the mediating effect of changes in body-mass index (BMI), alcohol, smoking, depressive symptoms and negative life events at follow-up. We observed less favorable changes in SBP (+1.12mmHg for women, +1.38mmHg for men), DBP (+0.85mmHg, +0.80mmHg) and FPG (only in women, +0.12 mmol/L) over time in the exposed group relative to the control group. Conversely, changes in HbA1c (−0.65 mmol/mol, −0.84 mmol/mol) and eGFR (+1.06 mL/min, +1.04 mL/min) were more favorable in the exposed compared to the control group, respectively. Changes in SBP, DBP, and FPG were partially mediated by changes in behavioral factors, in particular BMI and alcohol consumption. Concluding, the COVID-19 pandemic, in particular behavioral changes associated with restrictive lockdown measures, may have negatively affected several CVD risk factors, in both women and men.

## Highlights

• Several cardiovascular disease risk factors deteriorated due to the pandemic.
• This was seen in women and men across ethnic- and socioeconomic groups.
• Changes in BMI and alcohol use partially mediated the found effects.
• These findings may provide evidence for pandemic preparedness guidelines.

## 1 Introduction

The coronavirus disease 2019 (COVID-19) pandemic (Emanueli et al., 2020), including the disrupting effects of the measures taken to reduce the spread of the virus, such as lockdowns and social distancing measures, have been speculated to negatively impact risk factors for cardiovascular disease (CVD) (Lau & McAlister, 2021; Muhammad & Abubakar, 2021). For instance, studies have shown worse hypertension and diabetes control during the pandemic (Kolkailah et al., 2022; Lau & McAlister, 2021). Understanding how the pandemic affected cardiometabolic health could improve prevention and monitoring of high-risk groups during resurgence of the pandemic, and may aid decision making surrounding the management of future virus outbreaks.

These effects may in part be due to lower care use during the pandemic (Butt et al., 2022; Lau & McAlister, 2021), but also dietary changes, decreased exercise, more sedentary lifestyles and poorer wellbeing (Di Fusco et al., 2021; Kolkailah et al., 2022; Komiyama & Hasegawa, 2021; Lau & McAlister, 2021; Roth et al., 2022; Yazici et al., 2022), which in turn may have led to a deterioration in metabolic CVD risk factors (Kolkailah et al., 2022; Muhammad & Abubakar, 2021). As the pandemic is known to affect women and men differently, for example concerning women's and men's employment, psychological distress, and health behavior (Mattioli et al., 2022; Poelman et al., 2020; Vanderlind et al., 2021; Yerkes et al., 2020), we hypothesize this might translate into sex differences in the effect of the pandemic on metabolic CVD risk factors.

We studied the effect of the pandemic on temporal change in six metabolic CVD risk factors (Ramezankhani et al., 2017): systolic and diastolic blood pressure (SBP, DBP), total cholesterol (TC), fasting plasma glucose (FPG), Hemoglobin A1c (HbA1c), and estimated glomerular filtration rate (eGFR), in women and men aged 18–70 without prior CVD. On top of the effects of aging on these risk factors (Baba et al., 2015; Gurven et al., 2012), we expect exposure to the pandemic to be associated with greater short-term deteriorations in these risk factors, meaning greater increases in SBP, DBP, TC, FPG, HbA1c, and greater decreases in eGFR (Ramezankhani et al., 2017). Second, we explored to what extent effects were mediated by changes in health behavior, depressive symptoms, and negative life events, as we expect exposure to the pandemic to be associated with worse diets, and increased substance use, depressive symptoms and stress due to negative life events, which may negatively affect the metabolic CVD risk factors (Komiyama & Hasegawa, 2021; Kreutz et al., 2021).

## 2 Materials and methods

Our study is nested within the multi-ethnic population-based Healthy Life in an Urban Setting (HELIUS) study (Snijder et al., 2017). Baseline data were collected between 2011 and 2015 among 24,789 Dutch, South-Asian Surinamese, African Surinamese, Ghanaian, Moroccan, and Turkish origin women and men aged 18–70 years living in Amsterdam, the Netherlands. Potential participants were sampled at random from the municipality registry after stratification by country of birth (Stronks et al., 2009). The second wave of data collection took place between May 2019 and November 2022. Data were obtained by questionnaire and physical examinations (including biological samples). The questionnaire covered several topics, including socioeconomic factors, sociocultural factors, health behavior, and mental health. Questionnaires were available in Dutch, and in English or Turkish for those who were not proficient in Dutch. Moreover, an ethnically-matched interviewer was available to assist with filling out the questionnaire. Physical examinations were conducted by trained research assistants.

### 2.1 Study design

**Fig. 1:**
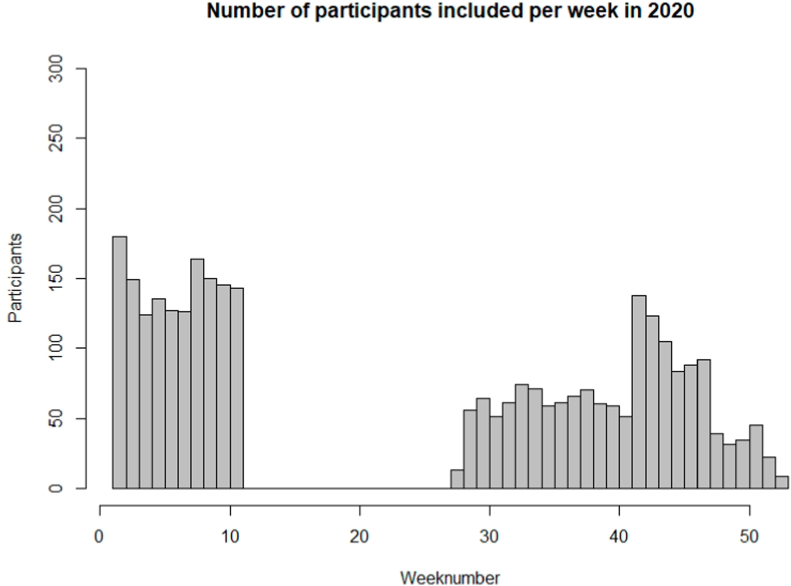
Number of participants included per week in 2020.

We conducted a natural experiment, comparing participants exposed to the pandemic during follow-up measurements (exposed group), and those not exposed (control group). This was possible because follow-up data collection was conducted partially before, and partially after the first lockdown (Yerkes et al., 2020) (Fig. 1). The control group consisted of participants whose follow-up measurements were taken between 15 May 2019 and 14 March 2020. The exposed group consisted of participants examined in a period with increasing infection rates and corresponding restrictive measures between 7 July 2020 and 30 December 2020. For a detailed description of the Dutch lockdown measures, we refer to Yerkes et al., 2020.

We included 8324 people who participated in the baseline measurements, and whose follow-up measurements were taken between May 2019 and December 2020. Those with unknown (N=21), Javanese (N=99) or other Surinamese ethnicity (N=118) were excluded due to low power. Next, those with prior CVD (N=1035) or missing data on CVD (n=89) were excluded. The final sample totaled 6962 participants: 5100 in the control group and 1862 in the exposed group.

#### 2.1.1 Metabolic CVD risk factors

All outcome variables were continuous: change over time in SBP, DBP, HbA1c, FPG, TC, and eGFR were calculated by subtracting baseline from follow-up values. The same data collection protocols and methods were used at baseline and follow-up (Snijder et al., 2017). SBP and DBP were measured in duplicate on the participants’ left arm using an automated digital blood pressure (BP) device after the participant had been sitting for 5 min. To determine FPG, HbA1c, TC and serum creatinine, fasting blood samples were drawn after an overnight fast. FPG was determined in plasma samples by using enzymatic spectrophotometric UV method, using hexokinase as primary enzyme (Roche Diagnostics), and HbA1c was determined in whole blood samples through HPLC technology (Tosoh). Serum creatinine was determined using a kinetic colorimetric spectrophotometric isotope dilution mass spectrometry-calibrated method (Roche Diagnostics), and eGFR was calculated using the CKDEPI (CKD epidemiology collaboration) creatinine equation (Levey & Stevens, 2010).

#### 2.1.2 Ethnicity

Ethnicity was defined by participants' and their parents' country of birth (Stronks et al., 2009). Participants were defined as belonging to one of the included minority groups if they, and at least one of their parents were born outside the Netherlands, or if they were born in the Netherlands, but both parents were born outside the Netherlands. Surinamese participants were further classified according to self-reported ethnic origin into ‘African’, ‘South-Asian’, ‘Javanese’, or ‘other’.

#### 2.1.3 Socioeconomic status

We used highest attained educational level, labor market participation, and occupational level at baseline as proxies for socioeconomic status (SES). Educational level was categorized into lower (no education, elementary education, or lower vocational or lower secondary education), intermediate (intermediate vocational, or intermediate or higher secondary education), or high (higher vocational education or university). Labor market participation was categorized into employed, not in employment (including retirees, students, homemakers), unemployed and/or social benefit recipients, and occupationally disabled. Occupational level was classified based on job title and description according to the Dutch Standard Occupational Classification system (Statistics Netherlands, 2010), and consisted of five categories: elementary, lower, intermediate, higher, and scientific occupations.

#### 2.1.4 Mediators

We considered negative life events at follow-up, and changes over time in BMI, smoking status, alcohol use, and depressive symptoms, as potential mediators. We selected these given that studies have reported unhealthier diets and weight gain (Bakaloudi et al., 2021; Barrea et al., 2020), higher rates of alcohol use (Bakaloudi et al., 2021), and smoking (Komiyama & Hasegawa, 2021; Kreutz et al., 2021), and more depressive symptoms (Pierce et al., 2020) as a result of the pandemic. Similarly, participants in the exposed group may have been more likely to report negative life events at follow-up compared to the control group, due to, e.g., losing a loved one due to the pandemic. Such changes in diet and BMI, alcohol use, smoking, depressive symptoms, and stress from such negative life events, have previously been associated with metabolic CVD risk factors (Komiyama & Hasegawa, 2021; Kreutz et al., 2021).

Changes in depressive symptoms and Body-Mass Index (BMI) were calculated by subtracting baseline from follow-up values. For BMI, weight and height were measured during physical examinations. Depressive symptoms, self-reported smoking status, alcohol use and negative life events were assessed via questionnaire at baseline and follow-up. Depressive symptoms were measured via the Patient Health Questionnaire (PHQ-9), a validated instrument (Galenkamp et al., 2017) measuring depressed mood in the past two weeks. Smoking status distinguished between current smokers, previous smokers and those who have never smoked, which was recoded into those who quit smoking, started smoking, or continued (not) smoking between baseline and follow-up. Alcohol use was classified as low, intermediate and high, which was recoded into decreased, equal or increased alcohol use between baseline and follow-up. Finally, negative life events at follow-up were defined based on whether people reported recently experiencing illness, death of a loved one, or other adverse experiences.

### 2.2 Data analysis

Sample characteristics were presented as means [standard deviations (SD)], or frequencies [percentages], by sex and control- or exposed group.

We tested associations between exposure to the pandemic, and change in metabolic risk factors, stratified by sex. We also tested the interaction between sex and exposure to the pandemic for each outcome, to study whether the pandemic differently affected women and men. We conducted these analyses using complete-case weighted linear regression analyses, using inverse probability weighting (IPW). IPW was used as selection bias and the phasing of data collection (i.e., for organizational reasons neighborhoods in Amsterdam were invited sequentially) could lead to baseline differences between the control and exposed group. This could impact our findings, as participants in the exposed group may have been more or less likely to see larger changes over time in these risk factors, compared to control group participants. Hence, in all analyses, we balanced the control and exposed group on baseline SBP, DBP, HbA1c, FPG, TC, and eGFR values, as well as baseline age, educational level, ethnicity, labor market participation, and occupational level. Weights were determined as (w=1/p(treated|x)) (Craig et al., 2017). Because we did not identify any large imbalances in the magnitudes of the weights after inspection, we refrained from using any stabilization methods (Chesnaye et al., 2022).

Next, we explored whether behavioral or psychosocial factors mediated the associations between exposure to the pandemic and change in metabolic risk factors. Specifically, we studied change in smoking status, alcohol use, BMI, and depressive symptoms, and negative life events at follow-up (Bakaloudi et al., 2021; Havranek et al., 2015; Kreutz et al., 2021; Virani et al., 2021). Additionally, as hyperfiltration may explain observed differences in eGFR, we explored whether changes in SBP and DBP mediated the association between the pandemic and changes in eGFR. Using weighted linear regression analyses, we compared the model without adjusting for our mediators to the model adjusted for all mediators, to measure whether these psychological and behavioral mechanisms contributed to the observed effects. Next, we adjusted for the mediators separately to see which contributed most to these changes. Decreases in beta's of ≥10% were considered indicative of mediation. Analyses were conducted in R studio 4.0.3, with p-values <.05 considered statistically significant.

### 2.3 Sensitivity analyses

Because medication use or seasonal effects might affect comparisons, we repeated our analyses, first, after excluding participants receiving HT and/or DM medication at baseline, and second, after restricting the control group to participants examined between July and December to ensure comparisons across similar time periods with the exposed group (Aubiniere-Robb et al., 2013). Next, we verified if effects were present across educational levels (as a proxy of SES) and ethnic groups, given that the pandemic may have differently affected ethnic and socioeconomic subgroups (Coyer et al., 2021; Kim et al., 2022; Pierce et al., 2020; Poelman et al., 2020; Yerkes et al., 2020).

### 2.4 Additional analysis

Next, we explored whether observed effects varied across time and the corresponding restrictive measures in place. We distinguished between an ‘early post-lockdown period’, between 7 July 2020, when data collection resumed, until 21 September 2020, reflecting a period with limited measures and low infection rates, and a ‘late post-lockdown period’ between 21 September until 30 December 2020, with new measures and increasing infection rates (Coyer et al., 2021).

Finally, we conducted a falsification analysis, to determine whether our findings may have been an artifact (Keele et al., 2019). We compared two groups within the control group: participants whose follow-up data collection took place between the start of follow-up data collection, before week 47 of 2019, and those whose follow-up data collection took place from week 47 of 2019 until the first lockdown.

## 3 Results

### 3.1 Sample characteristics

Of our participants, 56.2% was female. The mean age was 45.8 (SD=12.5) at baseline and 52.0 (SD=12.6) at follow-up, with an average follow-up time of 6 years and 2 months (Supplemental Table 1). Before weighting, the exposed group was younger, more often higher educated, and more often of Dutch origin than the control group (all p<.001). Baseline values for SBP, DBP, HbA1c (all p<.001), and FPG (p=.024), but not TC (p=.402) and eGFR (p=.056) were more favorable among exposed participants than control participants. After weighting, the pre- and post-pandemic group were more similar with regards to the sociodemographic factors and baseline CVD risk factors (Table 1). For instance, age in women and men was more comparable between the control group (47.2 [0.24] and 47.3 [0.26] years) and the exposed group (46.6 [0.44] and 47.0 [0.48]) than in the unweighted analyses.

**Table 1:** Weighted sociodemographic characteristics by sex and control versus exposed group.

	Women		Men	
	Control	Exposed	Control	Exposed
Mean follow-up time in months [SD]	73.4 [0.29]	79.5 [0.48]	72.4 [0.30]	78.1 [0.49]
Mean age at baseline [SD]	47.2 [0.24]	46.6 [0.44]	47.3 [0.26]	47.0 [0.48]

Ethnicity				
Dutch	359 [0.26]	697 [0.29]	330 [0.28]	[0.34]
South-Asian Surinamese	282 [0.20]	435 [0.18]	205 [0.17]	280 [0.14]
African Surinamese	413 [0.29]	558 [0.23]	240 [0.20]	415 [0.21]
Ghanaian	101 [0.07]	203 [0.09]	88 [0.07]	147 [0.07]
Turkish	91 [0.06]	198 [0.08]	115 [0.10]	192 [0.10]
Moroccan	155 [0.11]	295 [0.12]	196 [0.17]	280 [0.14]

Education				
Lower	514 [0.37]	824 [0.35]	439 [0.40]	684 [0.34]
Intermediate	434 [0.31]	694 [0.29]	345 [0.31]	585 [0.29]
High	451 [0.32]	868 [0.36]	319 [0.29]	732 [0.37]

Employment status				
Employed	1003 [0.72]	1727 [0.72]	904 [0.77]	1549 [0.77]
Not in employment	187 [0.13]	276 [0.12]	95 [0.08]	179 [0.09]
Unemployed/Social benefit recipient	143 [0.10]	245 [0.10]	125 [0.11]	211 [0.11]
Occupationally disabled	66 [0.05]	138 [0.06]	50 [0.04]	62 [0.03]

Occupational level				
Elementary	188 [0.13]	319 [0.13]	124 [0.11]	189 [0.09]
Lower	333 [0.24]	546 [0.23]	384 [0.33]	577 [0.29]
Intermediate	425 [0.30]	709 [0.30]	281 [0.24]	485 [0.24]
Higher	359 [0.26]	593 [0.25]	275 [0.23]	521 [0.26]
Scientific	94 [0.07]	219 [0.09]	110 [0.09]	229 [0.11]

Baseline metabolic CVD risk factors				
SBP	125.2 [0.37]	125.0 [0.77]	131.7 [0.36]	130.7 [0.68]
DBP	77.2 [0.21]	76.9 [0.41]	82.9 [0.22]	82.6 [0.42]
TC	5.08 [0.02]	5.03 [0.04]	5.02 [0.02]	5.08 [0.04]
FPG	5.23 [0.02]	5.26 [0.04]	5.60 [0.02]	5.55 [0.04]
HbA1c	38.7 [0.14]	38.5 [0.28]	38.8 [0.17]	38.5 [0.32]
eGFR	99.9 [0.35]	100.3 [0.60]	97.8 [0.35]	97.6 [0.61]

*Values are presented as n [%] for ethnicity, educational level, employment status and occupational level, and as means [SD] for follow-up time, age at baseline, and for the baseline metabolic CVD risk factors. SBP, systolic blood pressure; DBP, diastolic blood pressure; TC, total cholesterol; FPG, fasting plasma glucose; HbA1c, haemoglobin A1c; eGFR, estimated glomerular filtration rate; SD, standard deviation.

### 3.2 Differences control and exposed group

**Fig. 2:**
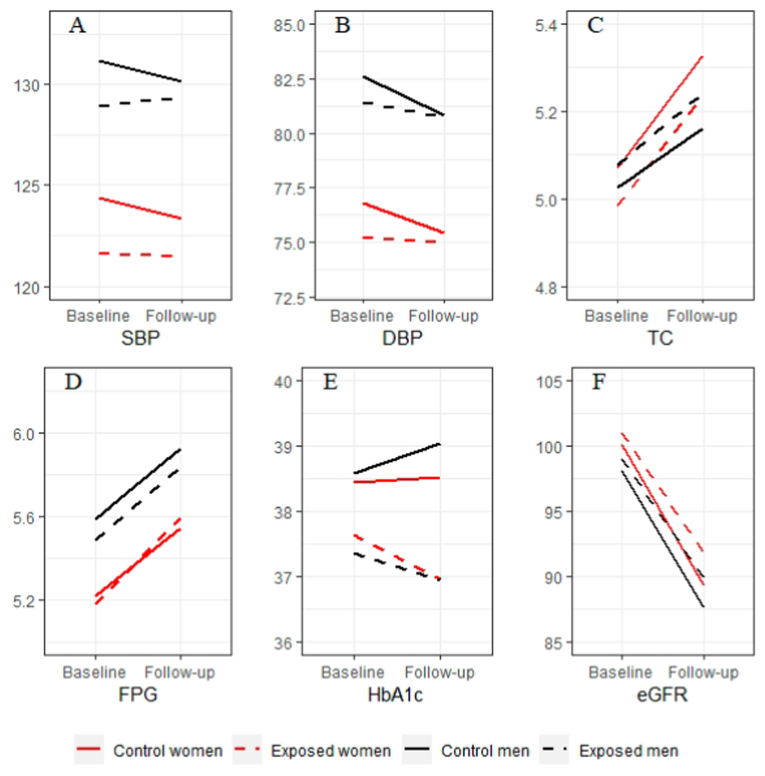
**Observed means of metabolic CVD risk factors at baseline and follow-up, in exposed- and control group women and men** *SBP, systolic blood pressure; DBP, diastolic blood pressure; TC, total cholesterol; FPG, fasting plasma glucose; HbA1c, hemoglobin A1c; eGFR, estimated glomerular filtration rate.

The exposed group experienced modestly larger increases in SBP, DBP, and FPG, smaller decreases in eGFR, and larger decreases in HBA1c than the control group (Supplemental Table 2). Change in TC was similar between groups. In weighted analyses, these differences remained (Table 2, Fig. 2). While the interaction was not statistically significant, exposed women appeared to have slightly greater deteriorations in DBP and FPG, and smaller improvements in HbA1c compared to exposed men, while exposed men appeared to have larger deteriorations in SBP and slightly smaller improvements in eGFR than exposed women.

**Table 2:** Differences in the weighted associations of exposure to the pandemic including lockdown measures, and temporal change in metabolic risk factors between women and men.

	Women		Men		Interaction	
	β [95% CI]	p-value	β [95% CI]	p-value	β [95% CI]	p-value
ΔSBP	1.12 [0.05, 2.19]	.041	1.38 [0.29, 2.47]	.013	-0.70 [-2.25, 0.85]	.377
ΔDBP	0.85 [0.23, 1.46]	.007	0.80 [0.11, 1.49]	.024	-0.04 [-0.97, 0.88]	.927
ΔTC	-0.05 [-0.12, 0.01]	.091	-0.01 [-0.07, 0.06]	.886	-0.05 [-0.15, 0.04]	.276
ΔFPG	0.12 [0.06, 0.19]	.000	0.02 [-0.07, 0.11]	.626	0.07 [-0.04, 0.18]	.189
ΔHbA1c	-0.65 [-0.97, −0.32]	.000	-0.84 [-1.37, −0.30]	.002	0.12 [-0.49, 0.72]	.712
ΔeGFR	1.06 [0.39, 1.74]	.002	1.04 [0.32, 1.76]	.005	-0.02 [-1.01, 0.96]	.963

SBP, systolic blood pressure; DBP, diastolic blood pressure; TC, total cholesterol; FPG, fasting plasma glucose; HbA1c, hemoglobin A1c; eGFR, estimated glomerular filtration rate; CI, confidence intervals. The analyses were weighted on baseline measurements, age at baseline, educational level, ethnicity, occupational level and labor market participation. The interaction column tests the interaction between exposure to the pandemic and lockdown and female sex.

### 3.3 Mediation analyses

The exposed group had more unfavorable changes in BMI, alcohol use (both p<.001), and smoking (p=.016) than the control group (Supplemental Table 3). Changes in these factors partially explained observed differences between the control and exposed group in several risk factors. For instance, in the model with all mediators, the beta's for the exposed versus control group for DBP decreased with 50.8% for women and 90% for men (Table 3). Changes in BMI and alcohol use, and smoking status for women, were associated with the largest decreases in beta's for SBP, DBP, and FPG (in men), but not HbA1c. Only change in alcohol use partially mediated the relationship between the pandemic and change in eGFR (−11.8% and −35.3% for men and women, respectively). As we found no changes in TC between the exposed and control group, we did not perform mediation analyses for TC. We found no differences in depressive symptoms and negative life events between the exposed and control group, and subsequently, we found no mediating effects of these psychological factors for any of the outcomes.

**Table 3:** Weighted associations of exposure to the pandemic, including lockdown measures, and temporal change in metabolic risk factors, adjusting for mediators.

	Women			Men		
	β [95% CI]	p-value	%Δ in β	β [95% CI]	p-value	%Δ in β
ΔSBP main analysis	1.119 [0.046, 2.193]	.041		1.380 [0.288, 2471]	.013	
+ Alcohol	0.962 [-0.277, 2.202]	.128	-14.0%	0.762 [-0.489, 2.013]	.233	-44.8%
+ Smoking	0.978 [-0.098, 2.053]	.075	-12.6%	1.357 [0.264, 2.450]	.015	-1.7%
+ BMI	0.787 [-0.270, 1.844]	.145	-29.7%	1.021 [-0.048, 2.089]	.061	-26.0%
+ PHQ-9	1.072 [-0.005, 2.150]	.051	-4.2%	1.320 [0.225, 2.415]	.018	-4.3%
+ NLE	1.090 [0.015, 2.166]	.047	-2.6%	1.380 [0.288, 2.471]	.013	0.0%
+ All mediators	0.428 [-0.800, 1.655]	.495	-69.1%	0.343 [-0.883, 1.568]	.583	-75.1%

ΔDBP main analysis	0.848 [0.232, 1.463]	.007		0.797 [0.107, 1.488]	.024	
+ Alcohol	0.712 [-0.001, 1.426]	.050	-16.0%	0.432 [-0.359, 1.223]	.284	-45.8%
+ Smoking	0.777 [0.161, 1.393]	.013	-8.4%	0.758 [0.067, 1.450]	.032	-4.9%
+ BMI	0.617 [0.017, 1.217]	.044	-27.2%	0.515 [-0.153, 1.182]	.131	-35.4%
+ PHQ-9	0.838 [0.220, 1.456]	.008	-1.2%	0.725 [0.032, 1.418]	.040	-9.0%
+ NLE	0.845 [0.229, 1.461]	.007	-0.4%	0.793 [0.103, 1.484]	.024	-0.5%
+ All mediators	0.417 [-0.282, 1.117]	.243	-50.8%	0.080 [-0.683, 0.843]	.837	-90.0%

ΔFPG main analysis	0.121 [0.056, 0.185]	.000		0.022 [-0.066, 0.109]	.626	
+ Alcohol	0.141 [0.066, 0.215]	.000	+16.5%	0.012 [-0.089, 0.112]	.820	-45.5%
+ Smoking	0.120 [0.055, 0.185]	.000	-0.8%	0.027 [-0.061, 0.114]	.554	+22.7%
+ BMI	0.110 [0.045, 0.174]	.001	-9.0%	0.013 [-0.075, 0.100]	.776	-40.9%
+ PHQ-9	0.127 [0.064, 0.190]	.000	+5.0%	0.019 [-0.070, 0.107]	.679	-13.6%
+ NLE	0.121 [0.056, 0.186]	.000	0.0%	0.022 [-0.066, 0.109]	.628	0.0%
+ All mediators	0.137 [0.065, 0.209]	.000	+13.2%	0.006 [-0.095, 0.107]	.908	-72.7%

ΔHbA1c main analysis	-0.645 [-0.974, −0.316]	.000		-0.835 [-1.370, −0.300]	.002	
+ Alcohol	-0.625 [-1.008, −0.242]	.001	-3.1%	-0.808 [-1.419, −0.197]	.010	-3.2%
+ Smoking	-0.641 [-0.972, −0.311]	.000	-0.6%	-0.795 [-1.330, −0.260]	.004	-4.8%
+ BMI	-0.731 [-1.056, −0.406]	.000	+13.3%	-0.892 [-1.427, −0.357]	.001	+6.8%
+ PHQ-9	-0.620 [-0.946, −0.295]	.000	-3.9%	-0.861 [-1.399, −0.323]	.002	+3.1%
+ NLE	-0.647 [-0.976, −0.317]	.000	+3.1%	-0.838 [-1.373, −0.304]	.002	+3.6%
+ All mediators	-0.678 [-1.053, −0.304]	.000	+5.1%	-0.821 [-1.434, −0.209]	.009	-1.7%

ΔeGFR main analysis	1.062 [0.387, 1.737]	.002		1.041 [0.322, 1.760]	.005	
+ Alcohol	0.687 [-0.088, 1.462]	.082	-35.3%	0.918 [0.096, 1.739]	.029	-11.8%
+ Smoking	1.065 [0.387, 1.743]	.002	+0.3%	1.070 [0.351, 1.788]	.004	+2.8%
+ BMI	1.110 [0.435, 1.785]	.001	+4.5%	1.193 [0.480, 1.907]	.001	+14.6%
+ PHQ-9	0.997 [0.320, 1.673]	.004	-6.1%	1.096 [0.374, 1.818]	.003	+5.3%
+ NLE	1.073 [0.399, 1.747]	.002	+1.0%	1.041 [0.323, 1.760]	.005	0.0%
+ SBP and DBP	1.025 [0.350, 1.700]	.003	-3.5%	1.015 [0.295, 1.735]	.006	-2.5%
+ All mediators	0.690 [-0.088, 1.468]	.082	-35.0%	1.121 [0.303, 1.939]	.007	+7.7%

Mediation for TC is not reported because TC did not differ between the control and exposed group in the main analyses. The analyses were weighted on baseline measurements, age at baseline, educational level, ethnicity, occupational level and labor market participation. SBP, systolic blood pressure; DBP, diastolic blood pressure; TC, total cholesterol; FPG, fasting plasma glucose; HbA1c, hemoglobin A1c; eGFR, estimated glomerular filtration rate; BMI, body mass index; PHQ, patient health questionnaire; NLE, negative life event; CI, confidence intervals.

### 3.4 Sensitivity and additional analyses

Analyses excluding participants receiving HT and/or DM treatment and analyses for seasonal effects did not change our interpretation (Supplemental Tables 4–5). Moreover, results on SBP, DBP and FPG, but not TC, HbA1c and eGFR, were similar in analyses stratified by ethnicity and SES, except for African Surinamese women and Turkish men. Yet, we have insufficient power to statistically assess whether changes in metabolic CVD risk factors varied by SES or ethnicity (Supplemental Tables 6–7).

Finally, the analysis across different time periods and phases of the pandemic revealed that associations between the exposed and control group in SBP, DBP, FPG and TC (the latter only in men), were larger in the late- compared to the early post-lockdown period (Supplemental Table 8).

The falsification analysis showed that the group included between week 47 and the lockdown had greater change over time in SBP and DBP, but no greater change in TC, FPG, HbA1c, and eGFR, compared to the group included before week 47 (Supplemental Table 9).

## 4 Discussion

Women and men in the exposed group experienced slightly greater increases in SBP, DBP and FPG than the control group. Change in TC did not differ between groups, while the exposed group experienced slightly more favorable changes in HbA1c and eGFR than the control group. Patterns did not vary significantly between women and men, and were observed across most ethnic and socioeconomic groups. Particularly in men, but also in women, changes in SBP, DBP, FPG and eGFR may have been partially mediated by factors related to health behavior, most notably changes in BMI and alcohol use.

While natural experiments are generally considered stronger than cross-sectional designs (Craig et al., 2017), and difference-in-differences analyses are frequently used with the aim to measure causal effects of ‘treatments’ (including policies, interventions or environmental hazards), some limitations persist (Richardson et al., 2023). First, possible response bias may have affected the generalizability of our study (Snijder et al., 2017). While we aimed to reduce response bias and baseline differences between the control and exposed group through IPW, we could not adjust for unmeasured confounders. Since propensity score matching would have negatively affected our power through loss of cases, IPW is our preferred method of adjustment (Sheikhy et al., 2021).

Second, we could not control for COVID-19 infections, as data on infections were not available. Hence, we cannot with certainty attribute effects to either COVID-19 infections or the lockdown measures. Moreover, we could not adjust for differences in follow-up time due to the pause in data collection, as this directly correlates with exposure to the pandemic. Differences in follow-up time may have affected variables that change with age, such as SBP and eGFR. Yet, this is unlikely to fully explain our findings, given that the difference in follow-up time is only a few months, while on average, SBP increases with 7 mmHg every ten years (Gurven et al., 2012) and eGFR decreases with a 1 mL/min/year rate (Baba et al., 2015). While the falsification analysis showed no association of timing of inclusion with TC, FPG, HbA1c, and eGFR, the association of timing with SBP and DBP appeared similar to the main analysis. While we may also have captured the effects of health behaviors during the holiday season on these metabolic risk factors, it implies that the findings for the observed effect of the lockdown in our main analyses may (in part) be related to other (contextual) factors than the pandemic and lockdown.

Furthermore, the effect of the mediators may have been underestimated, as measures may have been suboptimal, e.g., only change in smoking status was available, instead of amount and products smoked. Moreover, while we used the most common method of assessing these variables (Snijder et al., 2017), most mediators were self-reported, which may be less accurate compared to other methods (Hebert, 2016).

Despite these limitations, we found statistically significant differences in temporal changes in several metabolic CVD risk factors between the control and exposed group, suggesting a potential effect of the pandemic on future CVD risk in women and men. This is in line with literature reporting deteriorations in CVD risk factors and the management of CVD risk factors due to the pandemic (Kolkailah et al., 2022; Komiyama & Hasegawa, 2021; Lau & McAlister, 2021; Muhammad & Abubakar, 2021). The magnitude of these associations appears modest, yet, observed effects could be problematic at the population level. Associations were found across socioeconomic and ethnic groups, and density plots revealed a slight shift in the entire distribution of several risk factors (data not shown). Thus, in line with Rose's prevention paradox, this could imply a need for intervention strategies aimed at the total population rather than exclusively high-risk individuals (Raza et al., 2018).

While we expected larger increases in SBP and DBP in the exposed versus control group, results showed decreases in SBP in the control group, and decreases in DBP in the exposed- and control group. The overall decline over time may be due to treatment, as participants diagnosed with hypertension at baseline may have received treatment or lifestyle advice to lower their BP. However, these improvements in SBP and DBP were larger in the control group than the exposed group. This may be related to lower treatment adherence during the pandemic, for instance, patients with chronic disease may have been more likely to forget taking their medication (Ismail et al., 2022). Hence, our findings still suggest that, besides other contextual factors, the pandemic may have negatively affected BP.

We expected greater increases in FPG and HbA1c in the exposed group, yet, our findings on glycaemia were mixed. While we did find worse changes in FPG in exposed women, we also found more favorable changes in HbA1c in exposed women and men. Recent literature also shows mixed findings on the effect of the pandemic on glycaemia and glycemic control (Di Fusco et al., 2021; Ghosal et al., 2020; Kolkailah et al., 2022; Lau & McAlister, 2021; Yazici et al., 2022). A potential explanation for our contradictory findings on these different measures of glycaemia is that HbA1c, as a long-term measure of glycaemia, may be less accurate in capturing the acute effects of the pandemic (Rohlfing et al., 2002). This is supported by our finding that changes in HbA1c were worse in the late- compared to the early post-lockdown period.

We found no effect of exposure to the pandemic on increases in TC, despite the larger increase in BMI in the exposed group (Muga et al., 2019). This may be due to underlying dietary- and lifestyle patterns, which may associate more strongly with other cholesterol measures, such as HDL, LDL or triglycerides (Muga et al., 2019). Moreover, contrary to our expectations, the exposed group saw a smaller decline in eGFR than the control group, whereas we expected the pandemic to be associated with a greater decline in eGFR. This was not explained by hyperfiltration, as changes in BP did not mediate this association. Additionally, COVID-19 infections also likely do not explain this association, given that COVID-19 infections only cause acute kidney injury in a small proportion of infected individuals, and because Ghanaians, the group with the largest infection rate (Coyer et al., 2021), did not experience worse changes in eGFR. Alternatively, eGFR values may have been overestimated, as follow-up creatinine values may have been influenced by decreased muscle mass (Baxmann et al., 2008) due to decreased exercise during the lockdown (Di Fusco et al., 2021).

Associations between exposure to the pandemic and BP and glycaemia seem to be partially mediated by BMI and alcohol use. These findings are in line with studies on health behavior during the pandemic (Bakaloudi et al., 2021; Kolkailah et al., 2022), and indicate strategies promoting healthy lifestyles could be used in recovery following pandemics (Kolkailah et al., 2022). We found no effect of psychological mediators, despite studies reporting more mental distress during the pandemic (Kolkailah et al., 2022; Vanderlind et al., 2021). Possibly, the effects of the pandemic on mental health might not have been visible yet during this early phase of the pandemic, but have become more evident on the long term.

We found no sex differences in the effects of the pandemic, despite literature reporting unhealthier diets (Poelman et al., 2020) and more psychological problems (Mattioli et al., 2022; Vanderlind et al., 2021) in women, and worse sleeping patterns, decreased exercise (Barrea et al., 2020), higher alcohol consumption (Bakaloudi et al., 2021) and higher risk of DM (Ghosal et al., 2020) in men. While the pandemic may have affected women and men differently, this might not have translated into sex differences in the effect of exposure to the pandemic on CVD risk factors in the short term. Future research should determine whether this trend continues after the pandemic.

We conclude the pandemic may have led to modest deteriorations in metabolic CVD risk factors, in women and men across ethnic and socioeconomic groups. These effects seem to be partially mediated by changes in health behavior, likely as a result of the pandemic. This may be valuable in decision making surrounding the management (e.g., considering the implementation of lockdown measures) and recovery of the COVID-19 pandemic (such as preventive measures promoting healthy weight and reducing alcohol use), as well as future pandemics, and may provide evidence for pandemic preparedness guidelines.

## Ethnical approval

The HELIUS study has been approved by the AMC Ethical Review Board. All participants provided written informed consent.

## Funding

Dutch Heart Foundation (grant 2010T084), the Netherlands Organization for Health Research and Development (ZonMw; grant 200500003), the European Union (FP-7; grant 278901), and the European Fund for the Integration on non-EU immigrants (EIF; grant 2013EIF013). This work was additionally supported by the Netherlands Organization for Health Research and Development Gender and Health Program, and the Dutch Kidney Foundation (ZonMw, grant 849200008).

## Author statement

Bryn Hummel: conception and design of work, conducting analyses, drafting of manuscript.

Irene G. M. van Valkengoed: conception and design of work, critical revision of manuscript.

Mara A. Yerkes: critical revision of manuscript.

Ralf E. Harskamp: critical revision of manuscript.

Henrike Galenkamp: critical revision of manuscript.

Anton E. Kunst: critical revision of manuscript.

Anja Lok: critical revision of manuscript.

All authors gave final approval and agree to be accountable for all aspects of work ensuring integrity and accuracy. The corresponding author attests that all listed authors meet authorship criteria and that no others meeting the criteria have been omitted. All gave final approval and agree to be accountable for all aspects of work ensuring integrity and accuracy.

## Declaration of interest

None.

## Data Availability

The authors do not have permission to share data.
